# Carotid plaque inflammation and calcification on somatostatin receptor PET/CT imaging predict stroke and major adverse cardiovascular events

**DOI:** 10.1093/ehjci/jeag110

**Published:** 2026-04-28

**Authors:** Xiaoliang Shao, Carola Maria Bregenzer, Jiahui Wang, Yizhou Chen, Yuetao Wang, Luisa Maria Knappe, Axel Rominger, Kuangyu Shi, Federico Caobelli

**Affiliations:** Department of Nuclear Medicine, Inselspital, Bern University Hospital, University of Bern, Freiburgstrasse 18, Bern 3010, Switzerland; Department of Nuclear Medicine, the Third Affiliated Hospital of Soochow University, No. 185 Juqian Street, Tianning District, Changzhou, Jiangsu 213003, China; Department of Nuclear Medicine, Inselspital, Bern University Hospital, University of Bern, Freiburgstrasse 18, Bern 3010, Switzerland; Department of Nuclear Medicine, Inselspital, Bern University Hospital, University of Bern, Freiburgstrasse 18, Bern 3010, Switzerland; Department of Nuclear Medicine, Inselspital, Bern University Hospital, University of Bern, Freiburgstrasse 18, Bern 3010, Switzerland; Department of Nuclear Medicine, the Third Affiliated Hospital of Soochow University, No. 185 Juqian Street, Tianning District, Changzhou, Jiangsu 213003, China; Department of Nuclear Medicine, Inselspital, Bern University Hospital, University of Bern, Freiburgstrasse 18, Bern 3010, Switzerland; Department of Nuclear Medicine, Inselspital, Bern University Hospital, University of Bern, Freiburgstrasse 18, Bern 3010, Switzerland; Department of Nuclear Medicine, Inselspital, Bern University Hospital, University of Bern, Freiburgstrasse 18, Bern 3010, Switzerland; Department of Nuclear Medicine, Inselspital, Bern University Hospital, University of Bern, Freiburgstrasse 18, Bern 3010, Switzerland; Department of Nuclear Medicine, the Third Affiliated Hospital of Soochow University, No. 185 Juqian Street, Tianning District, Changzhou, Jiangsu 213003, China

**Keywords:** [^68^Ga]DOTA-TOC, carotid plaques, somatostatin receptor imaging, culprit plaques, cerebrovascular events

## Abstract

**Purpose:**

Carotid atherosclerosis is a risk factor for stroke and major adverse cardiovascular events (MACE) and is characterized by inflammation and calcification at different stages. Simultaneous assessment of both features may improve risk stratification. We therefore evaluated whether combined assessment of carotid plaque inflammation and calcification on [^68^Ga]DOTA-TOC PET/CT predicts stroke and MACE in a real-world oncologic population.

**Methods and results:**

We retrospectively included patients who underwent [^68^Ga]DOTA-TOC PET/CT for suspected or confirmed neuroendocrine tumours. Carotid plaques were assessed visually and semiquantitatively on PET/CT, and patients were categorized into four groups according to the patient-level presence of carotid artery calcification and/or focal [^68^Ga]DOTA-TOC uptake. Median follow-up was 55 months (IQR 42–72). Stroke and MACE, defined as myocardial infarction, hospitalization for myocardial ischaemia, coronary revascularization, and all-cause death, were recorded. A total of 353 patients were evaluated. Carotid artery calcification was present in 123/353 (34.8%) patients, with a median calcification volume of 77.7 cm^3^ (IQR 27.9–169.8). Forty-six patients (13.0%) showed 67 foci of [^68^Ga]DOTA-TOC uptake, with median SUVmax 1.68 (IQR 1.55–1.93) and median TBR 2.14 (IQR 1.65–2.79). Patients with both calcified and inflamed carotid plaques had the highest rates of stroke (15.2%) and MACE (12.1%) vs. all other groups (*P* < 0.05). In multivariable Cox analysis, this combined patient-level phenotype remained independently associated with stroke and MACE and remained significant in competing-risk analyses.

**Conclusion:**

[^68^Ga]DOTA-TOC PET/CT enables simultaneous assessment of macrophage-related carotid plaque inflammation and calcification and identifies a subgroup at particularly high risk of stroke and MACE.

## Graphical Abstract

**Graphical Abstract:**
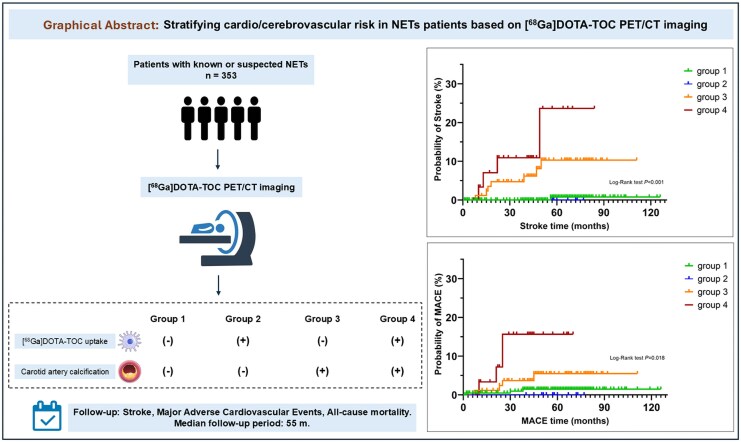

**See the editorial comment for this article ‘Is plaque inflammation the missing link for cardiovascular risk prediction?’, by M. Peverelli and J.M. Tarkin, https://doi.org/10.1093/ehjci/jeag137.**

## Background

Cardiovascular diseases remain the leading cause of death worldwide, accounting for nearly one-third of all global deaths.^1^ Among these, 81.8% are caused by ischaemic heart disease and stroke.^1,2^ Atherosclerotic plaques involving carotid arteries are not only a well-established risk factor for stroke but also a surrogate marker of systemic atherosclerosis and a predictor of major adverse cardiovascular events (MACE).^3^

Atherosclerosis is a complex, lipid-driven chronic inflammatory arterial disease whose progression is regulated by multiple factors and involves various pathological processes, such as vascular endothelial dysfunction, lipid deposition, inflammation and oxidative stress, and arterial calcification.^4^ A comprehensive evaluation of carotid plaque morphology, composition, and molecular characteristics can predict plaque stability and disease progression, providing significant value in risk stratification, prognosis prediction, and personalized clinical decision-making.^3,5^ Hence, non-invasive imaging of the carotid artery for early identification of high-risk features of atherosclerosis provides vital information for assessing stroke and overall cardiovascular risk.

Among numerous imaging targets, arterial calcification represents a characteristic feature of advanced atherosclerotic lesions and serves as a reliable indicator of overall plaque burden.^6^ In the PROSPECT study, total plaque burden was identified as the strongest predictor of MACE,^7^ although in the early stages of atherosclerosis, plaques may be non-calcified.^6^ On the other hand, active inflammation plays a key role in the vulnerability of vascular plaques and in the occurrence of complications, persisting throughout the entire course of the disease.^8^ As such, integrating information on both calcification and inflammation may enable a more accurate assessment of the severity of atherosclerosis, thereby further enhancing risk stratification.

In this regard, [^18^F]Fluorodeoxyglucose ([^18^F]FDG) positron emission tomography/computed tomography (PET/CT) is a sensitive modality to identify inflamed plaques, but lacks specificity in identifying activated macrophages.^9^ In contrast, somatostatin receptor imaging with [^68^Ga]DOTA-TOC PET/CT identifies expressed somatostatin receptor subtype 2 (SSTR2) on pro-inflammatory macrophages, offering a highly specific signal for plaque inflammation. Our previous research showed that patients with coronary artery calcifications and concomitant [^68^Ga]DOTA-TOC uptake had significantly higher rates of stroke and all-cause mortality compared to those without.^9^ Of note, our previous study was limited by a relatively small patient sample (*n* = 108) and a relatively short follow-up. It now remains unclear whether the presence of calcified and/or inflamed carotid plaques identified by [^68^Ga]DOTA-TOC PET/CT may yield similar prognostic value in the prediction of stroke and MACE. Therefore, the present study aimed to evaluate this in a real-world oncologic population with extended follow-up.

## Materials and methods

### Patient population

**Figure 1:**
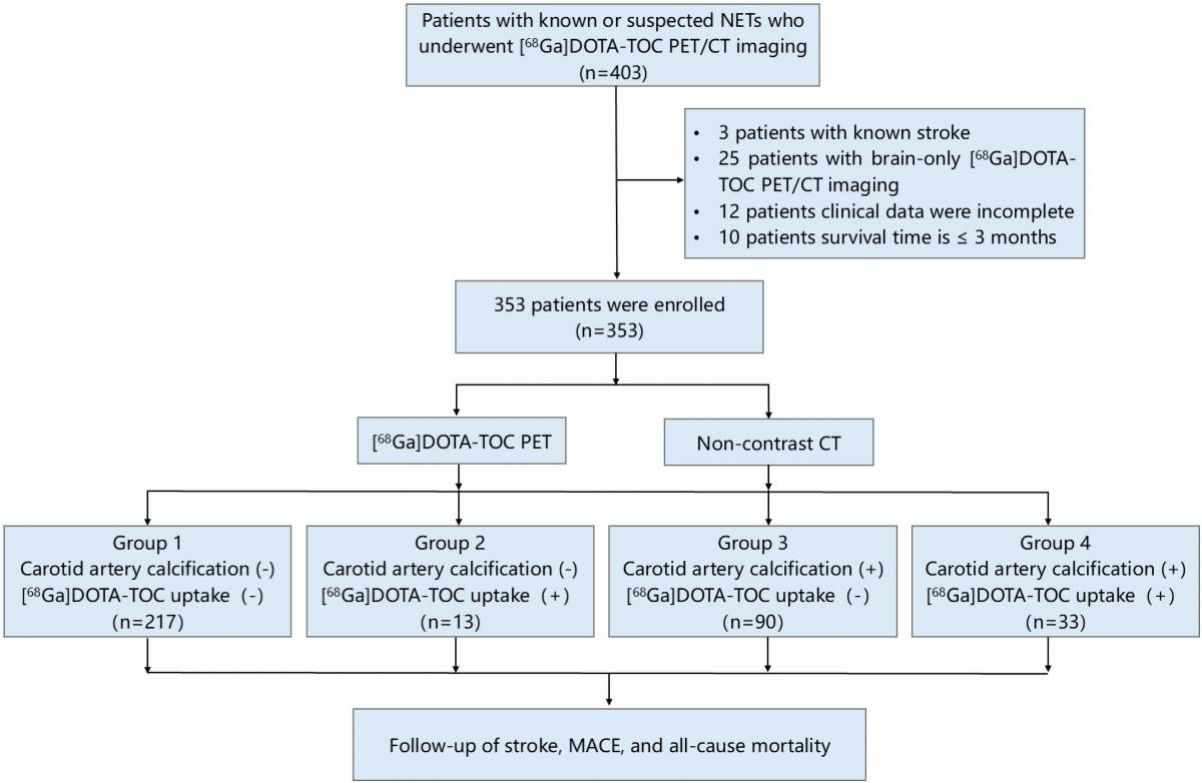
Flowchart of the present study.

We retrospectively analysed the complete record of patients with known or suspected neuroendocrine tumours (NETs) who underwent [^68^Ga]DOTA-TOC PET/CT imaging at the University Hospital of Bern, Switzerland, between June 2014 and July 2023. From these, we excluded patients with prior history of stroke, carotid artery surgery or stent implantation and neck radiation therapy. Patients undergoing brain-only [^68^Ga]DOTA-TOC PET/CT imaging, with incomplete clinical data records, poor image quality (significant metal artefacts or enlarged cervical lymph nodes) precluding quantitative analysis of carotid plaque or calcification were also excluded. If a patient had multiple [^68^Ga]DOTA-TOC PET/CT imaging, only the one performed first was considered. Baseline characteristics including cardio/cerebrovascular risk factors as well as oncologic data were recorded. The study was conducted according to the Declaration of Helsinki and was approved by the local Ethics Committee (KEK-Nr. 2022–00486). The outline of the present study is shown in *Figure 1*.

### Imaging protocol

Patients undergoing [^68^Ga]DOTA-TOC PET/CT did not require specific preparation. PET imaging was acquired 60 min after intravenous injection of 151.6 ± 14.0 MBq [^68^Ga]DOTA-TOC. The imaging equipment included standard axial field-of-view (SAFOV, Biograph mCT and Biograph Vision 600, Siemens Healthineers, Knoxville, TN, USA) or long-axial field-of-view (LAFOV, Biograph Vision Quadra Siemens Healthineers, Knoxville, TN, USA) PET/CT scanners. As previously published,^10^ images were acquired on the SAFOV scanner in continuous bed motion with a table velocity of 1.1 mm/s (skull-base to mid femur), equivalent to 2 min/bed position, while on LAFOV PET were acquired in list-mode for 10 min in a single bed position (skull-vertex to mid femur). The scanning and reconstruction parameters of CT images were performed as previously published.^10^ Non-contrast-enhanced, low-dose CT images were used for attenuation correction, identification, and to assess carotid artery calcification.

### Image evaluation

Anatomical coregistration of CT and PET was carefully verified prior to assessing [^68^Ga]DOTA-TOC uptake in the carotid arteries. Images were visually analysed slice-by-slice by two experienced nuclear medicine physicians who were blinded to clinical data. Lesions showing focal [^68^Ga]DOTA-TOC uptake visually above the surrounding background activity were additionally assessed semiquantitatively and considered PET-positive when the target-to-background ratio (TBR) was >1. A region of interest (ROI) was manually drawn on active foci in the carotid arteries to calculate maximum standardized uptake values (SUVmax). To normalize for blood pool activity, three standard ROIs (1 cm diameter) were placed in three consecutive slices in the superior vena cava, and SUVs were calculated as the mean value of the three measurements. TBR was defined as the ratio between SUVmax in the carotid artery and SUVmean of the blood pool.^3,11^ Visual analysis of carotid artery CT images was performed to assess calcification, defined as structures within the artery wall demonstrating a density greater than 130 HU.^12^ The calcification volume in bilateral carotid arteries was quantified by slice-by-slice delineation of ROI. To assess intra- and inter-reader reproducibility, the same reader and a second reader re-analysed the [^68^Ga]DOTA-TOC PET/CT images in a randomly selected sample of 100 cases >4 weeks after the initial assessment, and intraclass correlation coefficients (ICCs) with their 95% confidence intervals (CIs) were calculated.

### Follow up

Clinical records of stroke and MACE (myocardial infarction, hospitalization for myocardial ischaemia, coronary artery revascularization, all-cause death), were collected during the follow-up period. The time to the occurrence of MACE was recorded and defined as the time interval between [^68^Ga]DOTA-TOC and the first event. The median follow-up duration was 55 (interquartile range, 42–72) months.

### Statistical analysis

Statistical analysis was performed using SPSS software (version 30.0; IBM, USA). Normally distributed continuous variables are expressed as mean ± standard deviation (SD), and non-normally distributed data are reported as median with interquartile range (IQR; P25, P75). Categorical variables are presented as numbers and percentages (*n*, %). For group comparisons, the independent samples *t*-test was applied for normally distributed continuous variables, and the Mann–Whitney *U* test was used for non-normally distributed variables. Comparisons of categorical data were conducted using the chi-square test or Fisher’s exact test, as appropriate. Correlations between two continuous variables were examined using Pearson’s R. The rate of death and MACE was compared using Cox proportional hazard models to estimate hazard ratios (HRs) with 95% CIs and by Kaplan–Meier curves with Log-Rank test, dividing patients into four groups according to the presence of calcified and/or inflamed carotid plaques. Because of the limited number of events, parsimonious multivariable Cox models were used, and the covariates retained in the final models differed for stroke and MACE, as detailed in Supplementary data online, *Tables S1* and *S2*. Considering that tumour-related deaths may represent a competing risk for stroke and MACE, we further performed competing risk analyses using cumulative incidence functions and Fine-Gray sub-distribution hazard models. *P* values < 0.05 was considered statistically significant.

## Results

### Clinical characteristics

Of the 403 patients initially screened, 353 met the inclusion criteria. Of these, 94.3% (333/353) had a confirmed NETs. Baseline clinical characteristics are shown in *Table 1*.

**Table 1:** Baseline characteristics of study population (*n* = 353)

Characteristics	Value
Age (years)	60.3 ± 14.5
Male, *n* (%)	190 (53.8)
Body mass index (kg/m^2^)	25.8 ± 5.3
**Risk factors, *n* (%)**	
Overweight	113 (19.3)
Obesity	68 (32.0)
Current smokers	58 (16.4)
Family history of CVD	15 (4.2)
Hypercholesterolaemia	72 (20.4)
Hypertension	136 (38.5)
Diabetes mellitus	74 (21.0)
Peripheral artery disease	3 (0.8)
Prior cardiovascular events	16 (4.5)
**Tumour characteristics, *n* (%)**	
Known NETs	333 (94.3)
Small intestine	130 (36.8)
Pancreas	69 (19.5)
Colorectal	38 (10.8)
Lung	11 (3.1)
Others	85 (24.1)
Suspected NETs	20 (5.7)
PRRT therapy	70 (19.8)

PRRT, peptide receptor radionuclide therapy. Qualitative data: numbers (percentages); continuous data: mean ± SD.

### Relationship between [^68^Ga]DOTA-TOC uptake in the carotid arteries and calcification

One hundred twenty-three patients (34.8%) showed carotid artery calcification of any degree in at least one side, with a median calcification volume of 77.7 (27.9, 169.8) cm^3^. In 46 patients (13.0%), a total of 67 foci of increased [^68^Ga]DOTA-TOC uptake were detected in the carotid arteries (37 left, 30 right). The median SUVmax was 1.68 (1.55, 1.93), while the median TBR was 2.14 (1.65, 2.79). The intra-reader ICC for calcification volume and TBR measurements was 0.97 (95% CI: 0.95, 0.98) and 0.96 (95% CI: 0.93, 0.97), and the inter-reader ICC was 0.95 (95% CI: 0.94, 0.96) and 0.91 (95% CI: 0.86, 0.94).

Considering calcification and/or SSTR2 expression, 38.8% (26/67) of focal [^68^Ga]DOTA-TOC uptakes correlated with a detectable calcification on low-dose CT imaging. SUVmax [1.76 (1.47, 2.10) vs. 1.66 (1.57, 1.85), *P* = 0.837] and TBR [2.20 (1.53, 2.81) vs. 2.13 (1.71, 2.82), *P* = 0.842] were not different between calcified and non-calcified lesions.

**Figure 2:**
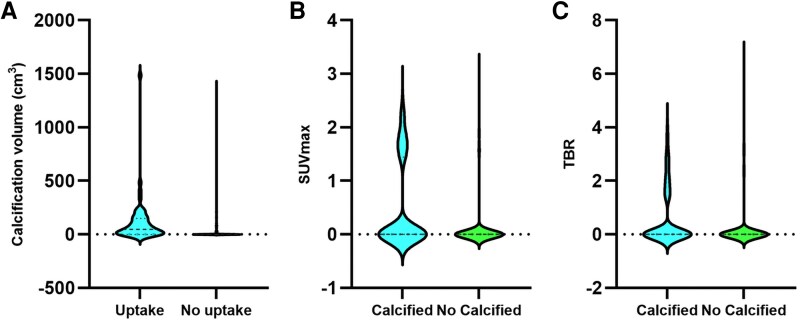
**Correlation between carotid artery calcification and focal [^68^Ga]DOTA-TOC uptake**. (*A*) Carotid artery calcification volume was significantly larger in patients with focal [^68^Ga]DOTA-TOC uptake than in those without, 47.2 (0, 149.1) cm^3^ vs. 0 (0, 13.4) cm^3^, *P* < 0.001; (*B,C*) [^68^Ga]DOTA-TOC uptake (SUVmax and TBR) was significantly higher in patients with carotid artery calcification than in those without, (*B*) SUVmax: 0 (0, 1.44) vs. 0 (0, 0), *P* < 0.001, (*C*) TBR: 0 (0, 1.38) vs. 0 (0, 0), *P* < 0.001.

As illustrated in *Figure 2*, patients with focal [^68^Ga]DOTA-TOC uptake demonstrated significantly greater carotid calcification volumes than those without uptake. And those with carotid calcification showed significantly higher [^68^Ga]DOTA-TOC uptake values (both SUVmax and TBR) compared to patients without calcification. Supporting this bidirectional relationship, calcification volume correlated moderately with both SUVmax (*r* = 0.322) and TBR (*r* = 0.310), with *P* < 0.001 for both.

### Relationship among carotid artery calcification, focal [^68^Ga]DOTA-TOC uptake, and cardiovascular risk factors

**Figure 3:**
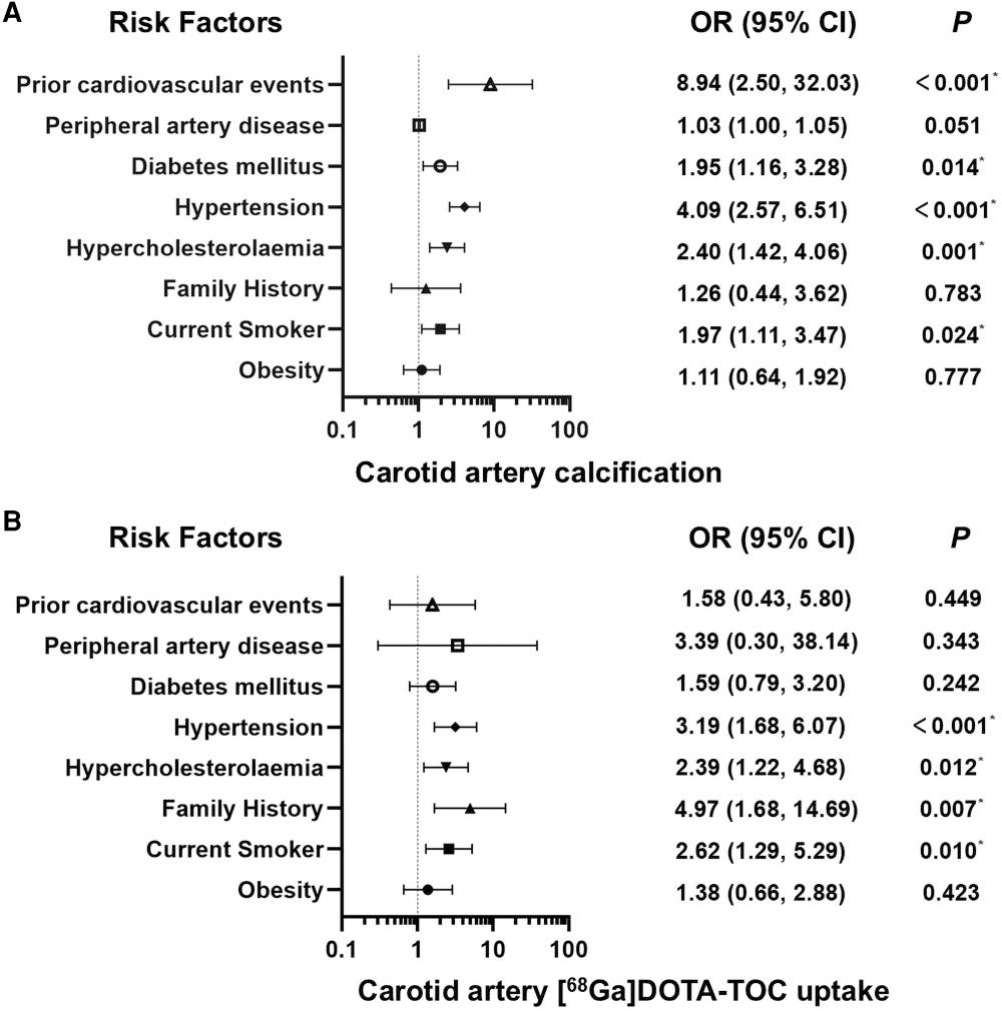
Relationship between cardiovascular risk factors and carotid artery calcification (*A*), focal [^68^Ga]DOTA-TOC uptake (*B*). * indicates a statistically significant.

As shown in *Figure 3*, smoking, hypercholesterolaemia, hypertension, diabetes, and prior cardiovascular events were correlated with higher rate of carotid artery calcification [odds ratio (OR): 1.95–8.94, *P* < 0.05, *Figure 3A*]. Further, a focal [^68^Ga]DOTA-TOC uptake in the carotid artery was more often seen in patients with smoking habits, family history of cardiovascular disease (CVD), hypercholesterolaemia, and hypertension (OR: 2.39–4.97, *P* < 0.05, *Figure 3B*).

Based on the patient-level presence of carotid artery calcification and focal [^68^Ga]DOTA-TOC uptake, patients were divided into four groups (Group 1: Calcification(-) and [^68^Ga]DOTA-TOC uptake(-); Group 2: Calcification(-) and [^68^Ga]DOTA-TOC uptake(+); Group 3: Calcification(+) and [^68^Ga]DOTA-TOC uptake(-); Group 4: Calcification(+) and [^68^Ga]DOTA-TOC uptake(+)). This classification reflects a patient-level imaging phenotype and does not imply that calcification and tracer uptake were colocalized within the same plaque. As shown in *Table 2*, patients with smoking, family history of CVD, hypercholesterolaemia, and hypertension showed a higher rate of both calcifications and focal [^68^Ga]DOTA-TOC uptake in the carotid arteries, consistent with inflamed and calcified plaques. If more risk factors are present (prevalence of ≥ 4 cardiovascular risk factors), this trend becomes even clearer (Group 1–4: 5.5%, 15.4%, 17.8%, and 24.2%, *P* = 0.002).

**Table 2:** Comparison of the proportion of patients with cardiovascular risk factors across groups (*n* = 353)

Risk factors	Group 1 (*n* = 217)	Group 2 (*n* = 13)	Group 3 (*n* = 90)	Group 4 (*n* = 33)	*P*
Obesity % (*n*)	17.1 (37)	46.2 (6)	22.2 (20)	15.2 (5)	0.053
Current smokers % (*n*)	12.4 (27)	23.1 (3)	18.9 (17)	33.3 (11)	0.018^a^
Family history of CVD % (*n*)	3.7 (8)	7.7 (1)	1.1 (1)	15.2 (5)	0.010^a^
Hypercholesterolaemia % (*n*)	14.7 (32)	23.1 (3)	26.7 (24)	39.4 (13)	0.004^a^
Hypertension % (*n*)	25.8 (56)	46.2 (6)	56.7 (51)	69.7 (23)	<0.001^a^
Diabetes mellitus % (*n*)	16.6 (36)	23.1 (3)	27.8 (25)	30.3 (10)	0.073
Peripheral artery disease % (*n*)	0 (0)	0 (0)	2.2 (2)	3.0 (1)	0.087
Prior cardiovascular events % (*n*)	1.4 (3)	0 (0)	11.1 (10)	9.1 (3)	<0.001^a^
High risk (≥ 4 risk factors) % (*n*)	5.5 (12)	15.4 (2)	17.8 (16)	24.2 (8)	<0.001^a^

Proportions among the four groups were compared using the chi-square or Fisher's test, with the *P*-value indicating the overall difference across the four groups.

^a^indicates a statistically significant *P*-value (*P* < 0.05).

### Risk of cardio/cerebrovascular events

**Figure 4:**
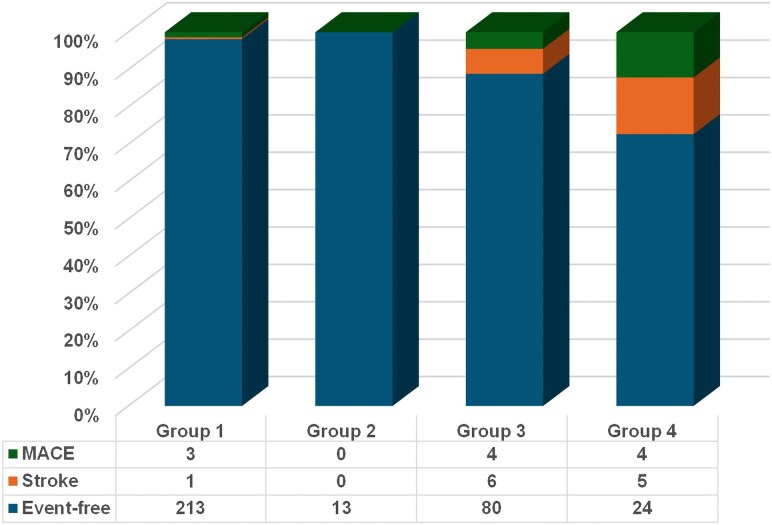
**The incidence of stroke and MACE in each group.** The incidence of stroke in Groups 1–4 was 0.5%, 0%, 6.7%, and 15.2%, respectively; χ^2^ = 19.090, *P* < 0.001. The incidence of MACE in Groups 1–4 was 1.4%, 0%, 4.4%, and 12.1%; χ^2^ = 9.241, *P* = 0.018.

**Figure 5:**
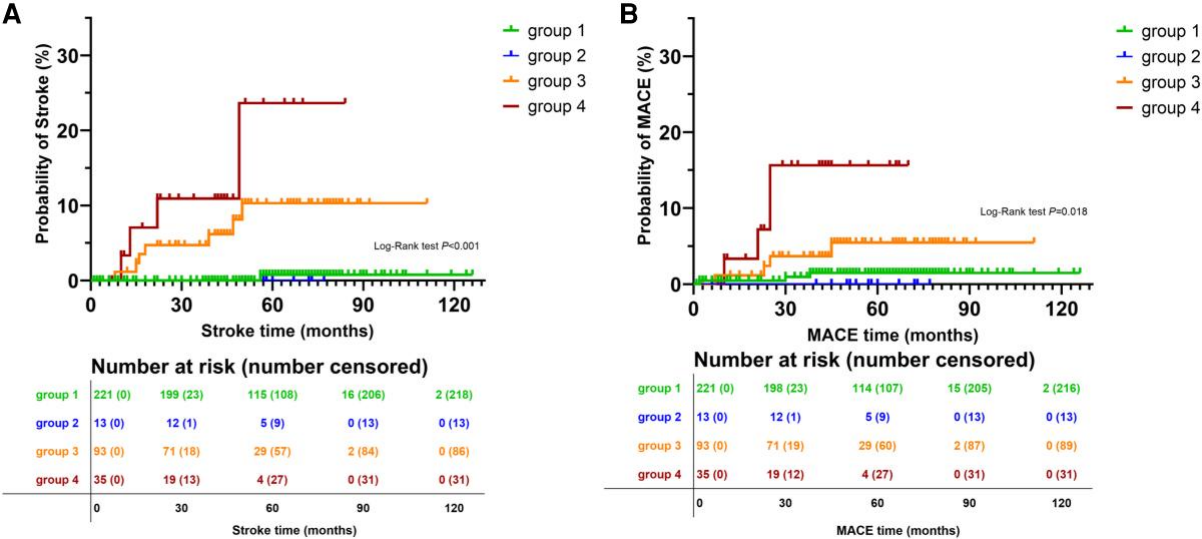
**Kaplan**–**Meier estimates of event probability for stroke (***A***) and MACE (***B***) across the four groups.** The presence of both carotid artery calcification and [^68^Ga]DOTA-TOC uptake was associated with a significantly higher risk of stroke and MACE compared to other presentations. Group 1: carotid artery calcification (−) [^68^Ga]DOTA-TOC uptake (−), Group 2: calcification (−) uptake (+), Group 3: calcification (+) uptake (−), Group 4: calcification (+) uptake (+).

During follow-up, non-fatal cardio/cerebrovascular events occurred in 23 patients (stroke: 12 patients, 3.4% and MACE: 11 patients, 3.1%), while 83 all-cause deaths (23.5%) were documented. The incidence of stroke and MACE in each group was as follows: Group 1: 0.5%, 1.4%; Group 2: 0%, 0%; Group 3: 6.7%, 4.4%; Group 4: 15.2%, 12.1% (χ^2^ = 19.090, 9.241; *P* < 0.001, 0.018, *Figure 4*). *Figure 5* shows KM-survival analysis demonstrating higher rate of MACE and stroke for patients in Group 4.

Univariate Cox regression analysis identified age, family history of CVD, hypertension, prior cardiovascular events, peripheral artery disease, the coexistence of carotid artery calcification, and [^68^Ga]DOTA-TOC uptake as risk factors for stroke (HR = 1.08–58.31, *P* all <0.05), smoking and the coexistence of carotid artery calcification and [^68^Ga]DOTA-TOC uptake were risk factors for MACE (*P* = 0.009 and <0.001). Multivariable COX analysis confirmed that the coexistence of carotid artery calcification and [^68^Ga]DOTA-TOC uptake was the independent risk factor for stroke (HR: 29.98, 95% CI: 3.22, 279.18, *P* = 0.017) and MACE (HR: 10.47, 95% CI: 2.24, 48.90, *P* = 0.003) in NETs patients (see Supplementary data online, *Tables S1* and *S2*). Through the competing risk analyses, the coexistence of carotid artery calcification and [^68^Ga]DOTA-TOC uptake remained significantly associated with both stroke (sub-distribution HR: 93.03, 95% CI: 9.47, 913.61, *P* < 0.01) and MACE (sub-distribution HR: 11.91, 95% CI: 2.42, 58.71, *P* < 0.01). In subgroup analysis, Group 4 had a significantly higher rate of both stroke and MACE compared to Group 3 (χ^2^ = 4.058, 4.397; *P* = 0.044, 0.036).

## Discussion

In our study, we demonstrated that the calcification burden within carotid plaques correlates with the presence of inflammation. Most importantly, the combined patient-level phenotype characterized by both carotid calcification and focal [^68^Ga]DOTA-TOC uptake was independently associated with stroke and MACE after adjustment for clinical risk factors, identifying a distinct subgroup at substantially elevated risk.

Atherosclerotic plaque formation and progression are driven by a multifactorial pathological process that evolves intermittently over time.^13^ Among the key cellular mediators of this process are pro-inflammatory macrophages, which play a central role in plaque progression and destabilization.^14^ When activated macrophages accumulate within the plaque, they may weaken the structural integrity of the fibrous cap, thereby increasing the risk of plaque rupture.^15^ In view of its high sensitivity in detecting inflammation, [^18^F]FDG PET/CT was firstly used to assess the prognostic impact of patients with inflamed carotid plaques. In a study by Moon *et al.*^16^ 1089 asymptomatic adults were followed up for 4.2 years, and 19 individuals (1.74%) developed cardiocerebrovascular events. On multivariable Cox regression, both carotid [^18^F]FDG uptake and intima-media thickness emerged as independent predictors of events (HR 2.98 and 2.82, respectively). These findings are directionally consistent with our results, although our study evaluated SSTR2-targeted inflammation imaging and additionally integrated plaque calcification assessment.

Additional carotid [^18^F]FDG studies provide important biological and clinical context for the interpretation of our findings. In a prospective cohort of 49 patients undergoing carotid endarterectomy, Cocker *et al*.^17^ showed that carotid [^18^F]FDG uptake correlated with plaque CD68 and CD45 expression and was higher in symptomatic than asymptomatic plaques, particularly in patients imaged within 90 days of a cerebrovascular event. Likewise, Giannotti *et al*.^18^ reported in 25 patients with recently symptomatic carotid stenosis that [^18^F]FDG uptake was positively associated with MRI markers of plaque vulnerability, including lipid-rich necrotic core, and inversely associated with fibrous cap thickness and calcium volume. Although these studies did not assess future stroke or MACE directly, they strengthen the biological plausibility of our observations by showing that increased carotid PET signal identifies plaques with inflammatory and morphological features of instability. Compared with these FDG-based studies, our data extend the literature by suggesting that SSTR2-targeted PET/CT, especially when combined with CT-based calcification assessment, may help identify a subgroup at particularly high risk of stroke and MACE. Prior studies also investigated the usefulness of other imaging modalities such as CT angiography^19^ or dual-tracer imaging using [^18^F]FDG and [^18^F]NaF^20^ in the assessment of inflammation and calcification in atherosclerotic plaques. However, it should be noted that perivascular fat attenuation indices derived from CT angiography offer only an indirect measure of inflammatory activity, and dual-tracer PET imaging typically requires a 2-day protocol and may cause higher radiation exposure.

Conversely, SSTR2 PET/CT imaging represents a more effective methodological approach for elucidating the interplay between inflammation and calcification, as already demonstrated by Rominger *et al*.,^11^ Malmberg *et al*.,^21^ and Mingels *et al*.^9^ In fact, SSTR2 PET/CT imaging can assess the presence of inflammation in target lesions, capitalizing on the expression of SSTR2 in activated macrophages. Tarkin *et al*.^22^ demonstrated that SSTR2 gene expression is closely associated with M1-like macrophages. In their work, strong correlations were seen between SSTR2 mRNA/CD68 mRNA levels and [^68^Ga]DOTA-TATE PET TBRmax in carotid plaques (*r* = 0.89 and 0.84, respectively), hence indicating that focal carotid uptake on SSTR2-targeted PET imaging directly reflects inflammatory activity and may represent a risk factor for cardio-cerebrovascular events.

In this regard, prior studies have explored the role of SSTR2 PET imaging in atherosclerosis, linking increased carotid or coronary plaque tracer uptake to conventional cardiovascular risk factors,^9,11,21^ or in the identification of high-risk vulnerable plaques,^22,23^ and in the assessment of the severity of cardiovascular disease.^24^ However, evidence on a direct association between SSTR2 PET findings and hard clinical endpoints such as stroke and MACE is still limited.^25^

Building upon our group's previous finding of increased stroke incidence in subjects with [^68^Ga]DOTA-TOC uptake in calcified coronary artery plaques,^9^ we now expanded on carotid plaques, also increasing sample size and follow-up duration.

While confirming that focal carotid [^68^Ga]DOTA-TOC uptake is associated with multiple cardiovascular risk factors (smoking, hypertension, hyperlipidaemia, and family history, etc.), our results suggest that the added prognostic value of PET lies in identifying, among patients with calcified carotid plaques, a subgroup at particularly high risk of stroke and MACE.

Calcification is a hallmark of advanced atherosclerotic disease,^26^ and the relationship between carotid calcification, plaque vulnerability, and clinical events remains debated. Carotid calcium scoring has been identified as an independent marker of carotid stenosis and ischaemic symptoms^12^ and predicts MACE and cardiovascular mortality following carotid endarterectomy.^26,27^ Yang *et al*.^28^ reported strong associations between carotid calcification (presence, multiplicity, superficial type) and intraplaque haemorrhage (OR = 3.0–3.9), a recognized feature of high-risk plaques and a biomarker for ischaemic cerebrovascular events.^29^ In a meta-analysis encompassing 89 studies and 22 683 patients, Bytyçi *et al*.^30^ demonstrated that carotid calcification and a higher calcified plaque burden are more prevalent among patients with significant coronary artery disease. However, the mere presence of calcification does not give precise information on plaque stability; rather, specific calcification characteristics, such as size, number, location, morphology, and composition, are more likely to provide useful markers to predict a worse outcome.^4,31,32^ A meta-analysis by Homssi^33^ found no significant link between carotid calcium burden/morphology and ischaemic stroke, while the ANTIQUE study^34^ reported a strong association of large carotid calcifications with coronary artery disease and atrial fibrillation. These inconsistent findings underscore the substantial heterogeneity of arterial calcified plaques, and a clear relationship between carotid calcification and stroke or MACE remains uncertain. In our study, incorporating the assessment of inflammation yielded to identify a subgroup of patients with even higher risk for adverse outcomes compared to patients with calcified, non-active plaques (15.2% vs. 6.7%). These findings seem to be in contrast with what has been reported by studies based on the analysis of coronary plaques, wherein microcalcifications and spotty calcifications (but not macrocalcifications) are more closely tied to vulnerable plaques and cardiovascular events.^19,35,36^ Differences in calcification patterns may arise from vascular-bed-specific variations in structure, cellular composition, molecular milieu, and haemodynamic forces.^37^ The fact that a strong association between macrocalcified carotid plaques and hard events could be found suggest that combined low-dose CT and [^68^Ga]DOTA-TOC PET imaging be used for assessment of cardio-cerebrovascular risk.

Intriguingly, our data suggest that isolated focal [^68^Ga]DOTA-TOC uptake without concomitant calcification does not predict a higher risk of stroke or MACE. From a pathophysiological perspective, inflammation is seen earlier in atherogenesis^13^ and inconsistently persists throughout its progression, whereas calcification may represent an endpoint resulting from sustained and excessive inflammation.^31,38^ The phenotypes of carotid plaque inflammation, calcification, and combined inflammation-calcification likely reflect distinct stages within the atherosclerotic continuum, being the ‘focal uptake only’ pattern suggestive of earlier disease. This is consistent with a study featuring CT coronary angiography and optical coherence tomography in 578 CAD patients, wherein Fujimoto *et al*.^19^ observed that vascular inflammation in early-to-intermediate atherosclerosis often occurs without concomitant calcification. During calcification accrual, lipid-rich plaques, macrophages, and microvessels initially increase before subsequently decreasing, indicating that the inflammation-calcification relationship is not merely linear. These insights may help explain the differential cardiovascular outcomes among phenotypic subgroups in our study and reinforce the concept of atherosclerosis as an intermittently progressing process characterized by cycles of ‘inflammation—healing—calcification—recurrent inflammation’.^13^ However, given the very small size of this subgroup, the lack of a statistically significant association may also reflect insufficient statistical power rather than a true negative finding. Accordingly, the prognostic significance of the PET(+)/CT(-) phenotype should be regarded as inconclusive rather than negative. Therefore, larger prospective studies with adequate sample sizes are needed to clarify the biological significance of isolated focal [^68^Ga]DOTA-TOC uptake without calcification, as well as its relationship with stroke and MACE.

### Limitations

Some limitations in our study should be acknowledged. First, this study has a retrospective design, and the studied population consisted of oncological patients with confirmed or suspected NETs, the mortality rate related to NETs is relatively high. Although we have conducted the competing risk analyses, the same results may not be completely transferred to a general population of disease-free patients.

Second, invasive or CT-based carotid angiography was not routinely performed to assess the degree of carotid stenosis. This represents a potential confounding factor that may be associated with a different rate of stroke and MACE. Moreover, as non-calcified, soft plaques were not systematically characterized with contrast enhanced CT, some focal [^68^Ga]DOTA-TOC uptakes without a corresponding CT lesion may be non-specific. Prior studies have reported an association between SSTR2-targeted tracer uptake and non-calcified coronary plaque features,^11,19^ thus we cannot exclude that part of the ‘uptake-only’ pattern in our cohort reflects non-calcified plaque substrate that was not detectable on low-dose CT, or alternatively non-specific uptake. This may have contributed to the lack of prognostic significance observed in patients with isolated [^68^Ga]DOTA-TOC uptake.

Third, the total number of stroke and MACE recorded was relatively low, reflecting the incidence rate of a population without known cerebrovascular disease. This limited the assessment of reliable thresholds for [^68^Ga]DOTA-TOC uptake parameters (such as SUVmax or TBR) or calcification volume to predict these events. Consequently, it was not feasible to construct a robust prognostic prediction model based on these quantitative plaque characteristics. Given the limited number of stroke and MACE events, the multivariable Cox models are at risk of overfitting and should be interpreted as exploratory. Furthermore, only 26 focal [^68^Ga]DOTA-TOC uptake sites in the carotid artery colocalized with carotid artery calcification, and due to the relatively small number of events, we were unable to explore the relationship between inflammatory calcified plaques and cardiovascular events at the lesion level.

Finally, the imaging examinations for the enrolled subjects were conducted over a considerable time span. During this period, the imaging instrumentation was upgraded. Differences in detection sensitivity and spatial resolution between the different scanner generations may introduce variability in the PET quantitative parameters.^9,10^

## Conclusions

Calcified carotid plaques represent a risk factor for the onset of cardio-cerebrovascular hard events. The combined patient-level presence of carotid calcification and M1-like macrophage-related inflammation as detected by [^68^Ga]DOTA-TOC PET/CT identifies a subgroup at particularly high risk of cardio-cerebrovascular hard events. Hence, [^68^Ga]DOTA-TOC PET/CT may be effectively used in order to plan an early start of an effective preventive therapy.

## Supplementary Material

jeag110_Supplementary_Data

## Data Availability

The data underlying this article will be shared on reasonable request to the corresponding author.

## References

[1] Global Burden of Cardiovascular Diseases and Risks 2023 Collaborators . Global, regional, and national burden of cardiovascular diseases and risk factors in 204 countries and territories, 1990-2023. J Am Coll Cardiol 2025;86:2167–243. 40990886 10.1016/j.jacc.2025.08.015

[2] MacAskill MG, Newby DE, Tavares AAS. Frontiers in positron emission tomography imaging of the vulnerable atherosclerotic plaque. Cardiovasc Res 2019;115:1952–62. 31233100 10.1093/cvr/cvz162 PMC6872971

[3] Saba L, Antignani PL, Gupta A, Cau R, Paraskevas KI, Poredos P et al International union of angiology (IUA) consensus paper on imaging strategies in atherosclerotic carotid artery imaging: from basic strategies to advanced approaches. Atherosclerosis 2022;354:23–40. 35816927 10.1016/j.atherosclerosis.2022.06.1014

[4] Saba L, Cau R, Vergallo R, Kooi ME, Staub D, Faa G et al Carotid artery atherosclerosis: mechanisms of instability and clinical implications. Eur Heart J 2025;46:904–21. 39791527 10.1093/eurheartj/ehae933

[5] Musialek P, Bonati LH, Bulbulia R, Halliday A, Bock B, Capoccia L et al Stroke risk management in carotid atherosclerotic disease: a clinical consensus statement of the ESC council on stroke and the ESC working group on aorta and peripheral vascular diseases. Cardiovasc Res 2025;121:13–43. 37632337 10.1093/cvr/cvad135

[6] Zheutlin AR, Chokshi AK, Wilkins JT, Stone NJ. Coronary artery calcium testing-too early, too late, too often. JAMA Cardiol 2025;10:503–9. 40042828 10.1001/jamacardio.2024.5644

[7] Stone GW, Maehara A, Lansky AJ, de Bruyne B, Cristea E, Mintz GS et al A prospective natural-history study of coronary atherosclerosis. N Engl J Med 2011;364:226–35. 21247313 10.1056/NEJMoa1002358

[8] Libby P, Soehnlein O. Inflammation in atherosclerosis: lessons and therapeutic implications. Immunity 2025;58:2383–401. 41045921 10.1016/j.immuni.2025.09.012

[9] Mingels C, Sari H, Gözlügöl N, Bregenzer C, Knappe L, Krieger K et al Long-axial field-of-view PET/CT for the assessment of inflammation in calcified coronary artery plaques with [(68) Ga]Ga-DOTA-TOC. Eur J Nucl Med Mol Imaging 2024;51:422–33. 37740742 10.1007/s00259-023-06435-6 PMC10774639

[10] Alberts I, Hünermund JN, Prenosil G, Mingels C, Bohn KP, Viscione M et al Clinical performance of long axial field of view PET/CT: a head-to-head intra-individual comparison of the biograph vision quadra with the biograph vision PET/CT. Eur J Nucl Med Mol Imaging 2021;48:2395–404. 33797596 10.1007/s00259-021-05282-7 PMC8241747

[11] Rominger A, Saam T, Vogl E, Ubleis C, la Fougère C, Förster S et al In vivo imaging of macrophage activity in the coronary arteries using 68Ga-DOTATATE PET/CT: correlation with coronary calcium burden and risk factors. J Nucl Med 2010;51:193–7. 20080898 10.2967/jnumed.109.070672

[12] Nandalur KR, Baskurt E, Hagspiel KD, Finch M, Phillips CD, Bollampally SR et al Carotid artery calcification on CT may independently predict stroke risk. AJR Am J Roentgenol 2006;186:547–52. 16423966 10.2214/AJR.04.1216 PMC2955288

[13] Libby P . Inflammation during the life cycle of the atherosclerotic plaque. Cardiovasc Res 2021;117:2525–36. 34550337 10.1093/cvr/cvab303 PMC8783385

[14] Gianopoulos I, Daskalopoulou SS. Macrophage profiling in atherosclerosis: understanding the unstable plaque. Basic Res Cardiol 2024;119:35–56. 38244055 10.1007/s00395-023-01023-z

[15] Barrett TJ . Macrophages in atherosclerosis regression. Arterioscler Thromb Vasc Biol 2020;40:20–33. 31722535 10.1161/ATVBAHA.119.312802 PMC6946104

[16] Moon SH, Cho YS, Noh TS, Choi JY, Kim BT, Lee KH. Carotid FDG uptake improves prediction of future cardiovascular events in asymptomatic individuals. JACC Cardiovasc Imaging 2015;8:949–56. 26189117 10.1016/j.jcmg.2015.06.002

[17] Cocker MS, Spence JD, Hammond R, deKemp RA, Lum C, Wells G et al [18f]-fluorodeoxyglucose PET/CT imaging as a marker of carotid plaque inflammation: comparison to immunohistology and relationship to acuity of events. Int J Cardiol 2018;271:378–86. 30007487 10.1016/j.ijcard.2018.05.057

[18] Giannotti N, McNulty J, Foley S, McCabe J, Barry M, Crowe M et al Association between 18-FDG positron emission tomography and MRI biomarkers of plaque vulnerability in patients with symptomatic carotid stenosis. Front Neurol 2021;12:731744. 35002912 10.3389/fneur.2021.731744 PMC8732361

[19] Fujimoto D, Kinoshita D, Suzuki K, Niida T, Yuki H, McNulty I et al Relationship between calcified plaque burden, vascular inflammation, and plaque vulnerability in patients with coronary atherosclerosis. JACC Cardiovasc Imaging 2024;17:1214–24. 39243232 10.1016/j.jcmg.2024.07.013

[20] Patil S, Kata R, Teichner E, Subtirelu R, Ghonim M, Ghonim M et al Associations of subclinical microcalcification and inflammation with carotid atheroma development: a dual-tracer PET/CT study. Eur J Nucl Med Mol Imaging 2025;52:2502–12. 39939531 10.1007/s00259-025-07127-z PMC12119761

[21] Malmberg C, Ripa RS, Johnbeck CB, Knigge U, Langer SW, Mortensen J et al 64Cu-DOTATATE for noninvasive assessment of atherosclerosis in large arteries and its correlation with risk factors: head-to-head comparison with 68Ga-DOTATOC in 60 patients. J Nucl Med 2015;56:1895–900. 26429961 10.2967/jnumed.115.161216

[22] Tarkin JM, Joshi FR, Evans NR, Chowdhury MM, Figg NL, Shah AV et al Detection of atherosclerotic inflammation by (68)Ga-DOTATATE PET compared to [(18)F]FDG PET imaging. J Am Coll Cardiol 2017;69:1774–91. 28385306 10.1016/j.jacc.2017.01.060 PMC5381358

[23] Pedersen SF, Sandholt BV, Keller SH, Hansen AE, Clemmensen AE, Sillesen H et al 64Cu-DOTATATE PET/MRI for detection of activated macrophages in carotid atherosclerotic plaques: studies in patients undergoing endarterectomy. Arterioscler Thromb Vasc Biol 2015;35:1696–703. 25977567 10.1161/ATVBAHA.114.305067 PMC4479665

[24] Jensen JK, Madsen JS, Jensen MEK, Kjaer A, Ripa RS. [(64)Cu]cu-DOTATATE PET metrics in the investigation of atherosclerotic inflammation in humans. J Nucl Cardiol 2023;30:986–1000. 36045250 10.1007/s12350-022-03084-4 PMC10261263

[25] Vergallo R, Park SJ, Stone GW, Erlinge D, Porto I, Waksman R et al Vulnerable or high-risk plaque: a JACC: cardiovascular imaging position statement. JACC Cardiovasc Imaging 2025;18:709–40. 40019413 10.1016/j.jcmg.2024.12.004

[26] Lucci C, Rissanen I, van den Beukel TC, Takx R, de Jong PA, Hendrikse J et al Risk factors for medial and intimal intracranial internal carotid artery calcification in men and women with cardiovascular disease: the UCC-SMART study. Cerebrovasc Dis 2024;53:734–42. 38286124 10.1159/000536422 PMC11633869

[27] Koek MMC, D'Oria M, Röder F, Bokkers RPH, Pol RA, Uyttenboogaart M et al Carotid calcium burden and perivascular fat density on computed tomography angiography as predictors of outcomes after carotid endarterectomy. J Vasc Surg 2025;82:2079–89. 40850608 10.1016/j.jvs.2025.08.023

[28] Yang J, Pan X, Zhang B, Yan Y, Huang Y, Woolf AK et al Superficial and multiple calcifications and ulceration associate with intraplaque hemorrhage in the carotid atherosclerotic plaque. Eur Radiol 2018;28:4968–77. 29876705 10.1007/s00330-018-5535-7 PMC6223859

[29] Bos D, Arshi B, van den Bouwhuijsen QJA, Ikram MK, Selwaness M, Vernooij MW et al Atherosclerotic carotid plaque composition and incident stroke and coronary events. J Am Coll Cardiol 2021;77:1426–35. 33736825 10.1016/j.jacc.2021.01.038

[30] Bytyçi I, Shenouda R, Wester P, Henein MY. Carotid atherosclerosis in predicting coronary artery disease: a systematic review and meta-analysis. Arterioscler Thromb Vasc Biol 2021;41:e224–37. 33626907 10.1161/ATVBAHA.120.315747

[31] Jebari-Benslaiman S, Galicia-García U, Larrea-Sebal A, Olaetxea JR, Alloza I, Vandenbroeck K et al Pathophysiology of atherosclerosis. Int J Mol Sci 2022;23:3346. 35328769 10.3390/ijms23063346 PMC8954705

[32] Tziotziou A, Fontana F, Korteland SA, Bierens J, Nederkoorn PJ, de Jong PA et al Carotid calcification shape, size, and lumen proximity are associated with ischemic events. Acad Radiol 2025;32:5468–77. 40562674 10.1016/j.acra.2025.05.066

[33] Homssi M, Saha A, Delgado D, RoyChoudhury A, Thomas C, Lin M et al Extracranial carotid plaque calcification and cerebrovascular ischemia: a systematic review and meta-analysis. Stroke 2023;54:2621–8. 37638399 10.1161/STROKEAHA.123.042807 PMC10530110

[34] Pakizer D, Šalounová D, Školoudík D. Extracranial carotid plaque calcification and its association with risk factors for cerebrovascular events: insights from the ANTIQUE study. Front Neurol 2025;16:1532883. 39944551 10.3389/fneur.2025.1532883 PMC11813772

[35] Homssi M, Vora A, Zhang C, Baradaran H, Kamel H, Gupta A. Association between spotty calcification in nonstenosing extracranial carotid artery plaque and ipsilateral ischemic stroke. J Am Heart Assoc 2023;12:e028525. 37183863 10.1161/JAHA.122.028525 PMC10227294

[36] Bhakta S, Tarkin JM, Chowdhury MM, Rudd JH, Warburton EA, Evans NR. Carotid atherosclerotic plaque microcalcification is independently associated with recurrent neurovascular events: a pilot study. Int J Stroke 2024;19:1155–61. 38888039 10.1177/17474930241264734 PMC11590389

[37] Soehnlein O, Lutgens E, Döring Y. Distinct inflammatory pathways shape atherosclerosis in different vascular beds. Eur Heart J 2025;46:3261–72. 40036569 10.1093/eurheartj/ehaf054 PMC12401584

[38] Nakahara T, Dweck MR, Narula N, Pisapia D, Narula J, Strauss HW. Coronary artery calcification: from mechanism to molecular imaging. JACC Cardiovasc Imaging 2017;10:582–93. 28473100 10.1016/j.jcmg.2017.03.005

