# Supplementary material for: Carotid plaque inflammation and calcification on somatostatin receptor PET/CT imaging predict stroke and major adverse cardiovascular events
Source: Eur Heart J Cardiovasc Imaging. 2026 Apr 28;27(8):1526–35. doi: 10.1093/ehjci/jeag110 (PMC13423501; doi:10.1093/ehjci/jeag110)
Supplement: jeag110_Supplementary_Data [file jeag110_supplementary_data.docx]

**Supplementary Table 1.** Univariate and multivariable Cox regression analyses of risk factors for stroke in NETs patients (n=353)

|  | **Univariate analysis** | | **Multivariate analysis** | |
| --- | --- | --- | --- | --- |
|  | *HR (95% CI)* | *P* | *HR (95% CI)* | *P* |
| **Age** | 1.08 (1.02, 1.15) | 0.010 |  |  |
| **Male** | 2.75 (0.74, 10.15) | 0.129 |  |  |
| **BMI** |  |  |  |  |
| **Normal** | 1 (Reference) |  |  |  |
| **Overweight** | 2.26 (0.64, 7.99) | 0.208 |  |  |
| **Obesity** | 1.23 (0.23, 6.71) | 0.812 |  |  |
| **Smoking** | 1.67 (0.45, 6.17) | 0.441 |  |  |
| **Family history of CVD** | 4.86 (1.07, 22.21) | 0.041 |  |  |
| **Hypercholesterolemia** | 3.02 (0.96, 9.50) | 0.060 |  |  |
| **Hypertension** | 8.92 (1.95, 40.81) | 0.005 | 5.79 (0.99, 34.03) | 0.052 |
| **Diabetes mellitus** | 0.35 (0.05, 2.74) | 0.319 |  |  |
| **Prior cardiovascular events** | 5.47 (1.19, 25.21) | 0.029 |  |  |
| **Peripheral artery disease** | 14.77 (1.90, 114.65) | 0.010 | 19.54 (1.70, 224.80) | 0.017 |
| **Imaging phenotype** | | | | |
| Group 1, 2 | 1 (Reference) |  | 1 (Reference) |  |
| Group 3 | 18.45 (2.21, 153.79) | 0.007 | 9.96 (1.15, 86.56) | 0.037 |
| Group 4 | 58.31 (6.69, 508.87) | <0.001 | 29.98 (3.22, 279.18) | 0.017 |

Note: BMI: body mass index, CVD: cardiovascular disease, Group 1: Calcification(-) and [68Ga]DOTA-TOC uptake(-); Group 2: Calcification(-) and [68Ga]DOTA-TOC uptake(+); Group 3: Calcification(+) and [68Ga]DOTA-TOC uptake(-); Group 4: Calcification(+) and [68Ga]DOTA-TOC uptake(+), * indicates a statistically significant P-value (P < 0.05)

**Supplementary Table 2.** Univariate and multivariable Cox regression analyses of risk factors for MACE in NETs patients (n=353)

|  | **Univariate analysis** | | **Multivariate analysis** | |
| --- | --- | --- | --- | --- |
|  | *HR (95% CI)* | *P* | *HR (95% CI)* | *P* |
| **Age** | 1.04 (0.99, 1.09) | 0.158 |  |  |
| **Male** | 4.08 (0.88, 18.90) | 0.072 |  |  |
| **BMI** |  |  |  |  |
| **Normal** | 1 (Reference) |  |  |  |
| **Overweight** | 1.01 (0.28, 3.57) | 0.991 |  |  |
| **Obesity** | 0.40 (0.05, 3.30) | 0.812 |  |  |
| **Smoking** | 4.24 (1.30, 13.90) | 0.017 | 2.97 (0.88, 10.00) | 0.078 |
| **Family history of CVD** | 2.27 (0.29, 17.72) | 0.435 |  |  |
| **Hypercholesterolemia** | 2.41 (0.70, 8.22) | 0.162 |  |  |
| **Hypertension** | 1.42 (0.43, 4.65) | 0.563 |  |  |
| **Diabetes mellitus** | 1.48 (0.39, 5.56) | 0.566 |  |  |
| **Prior cardiovascular events** | 0.00 (0.00, -) | 0.998 |  |  |
| **Peripheral artery disease** | 0 (0.00, -) | 0.998 |  |  |
| **Imaging phenotype** | | | | |
| Group 1, 2 | 1 (Reference) |  | 1 (Reference) |  |
| Group 3 | 3.72 (0.83, 16.63) | 0.086 | 3.30 (0.73, 14.90) | 0.120 |
| Group 4 | 13.35 (2.96, 60.33) | 0.001 | 10.47 (2.24, 48.90) | 0.003 |

Note: BMI: body mass index, CVD: cardiovascular disease, Group 1: Calcification(-) and [^68^Ga]DOTA-TOC uptake(-); Group 2: Calcification(-) and [^68^Ga]DOTA-TOC uptake(+); Group 3: Calcification(+) and [^68^Ga]DOTA-TOC uptake(-); Group 4: Calcification(+) and [^68^Ga]DOTA-TOC uptake(+), ^*^ indicates a statistically significant *P*-value (*P* < 0.05)
